# Offering within-category food swaps to reduce energy density of food purchases: a study using an experimental online supermarket

**DOI:** 10.1186/s12966-015-0241-1

**Published:** 2015-06-25

**Authors:** Suzanna E. Forwood, Amy L. Ahern, Theresa M. Marteau, Susan A. Jebb

**Affiliations:** Behaviour and Health Research Unit, Institute for Public Health, Forvie Site, University of Cambridge, Robinson Way, CB2 0SR Cambridge, UK; MRC Human Nutrition Research, Cambridge, UK; Nuffield Department of Primary Care Health Sciences, University of Oxford, Oxford, UK

**Keywords:** Food choice, Prompt, Healthy food, Grocery shopping

## Abstract

**Background:**

Swaps are often used to encourage healthier food choices, but there is little evidence of their effectiveness. The current study assessed the impact of offering swaps on groceries purchased within a bespoke online supermarket; specifically the objective was to measure the impact on energy density (ED) of food purchases following the offer of lower ED alternatives (a) at point of selection or at checkout, and (b) with or without explicit consent to receive swap prompts.

**Method:**

Participants were asked to complete a 12-item shopping task within an online shopping platform, developed for studying food purchasing. 1610 adults were randomly assigned to a no swap control condition or to one of four interventions: consented swaps at selection; consented swaps at checkout; imposed swaps at selection; or imposed swaps at checkout. Each swap presented two lower ED options from the same category as the participant’s chosen food. Swap acceptance rate and purchased food ED were the primary outcomes.

**Results:**

Of the mean 12.36 (SD 1.26) foods purchased, intervention participants were offered a mean of 4.1 (SD 1.68) swaps, with the potential to reduce the ED of purchased food (effect (95 % CI): −83 kJ/100 g (−110 – -56), p = <0.0001). A median of one swap (IQR 0 to 2) was accepted, not significantly reducing the purchased food ED (effect (95 % CI): −24 kJ/100 g (4 – -52), p = 0.094). More swaps were accepted when offered at selection than at checkout (OR (95 % CI) = 1.224 (1.11 – 1.35), *p* < 0.0001), but no differences were seen with consent. Purchased food ED was unaffected by point of swap or consent, but reduced with number of swaps accepted (effect per swap (95 % CI) = −24 kJ/100 g (−35 – -14), *p* < 0.0001).

**Conclusion:**

Within category swaps did not reduce the ED of food purchases reflecting the observation that the use of swaps within an on-line shopping platform offered small potential gains in ED and a minority was accepted.

**Electronic supplementary material:**

The online version of this article (doi:10.1186/s12966-015-0241-1) contains supplementary material, which is available to authorized users.

## Introduction

Swaps offering consumers the opportunity to replace their usual food with a healthier alternative are used as part of social marketing campaigns (e.g. Smart Swaps in the Change4Life campaign, UK). They have the potential to reduce the energy, fat, sugar or salt content of the diet because of the range of nutrient composition seen in many foods, particularly in categories that have undergone reformulation by some manufacturers. Swaps are already being used in the UK to alert consumers to cheaper alternatives (www.mysupermarket.com), so using swaps to promote health would be a scalable and low-cost intervention, but there is limited evidence on their effectiveness.

Swaps consisting of written lists of healthier foods as suggested swaps for intended purchases [1] or the previous month’s purchases [2] have been evaluated within multicomponent interventions [1–3] but the findings were inconclusive [1, 2]. One recent study [4] successfully adapted a real online supermarket to assess the impact of offering swaps at checkout targeted at lowering saturated fat, and showed a reduction in total saturated fat purchased of 10 %. To extend this finding further, the current study aims to assess the effectiveness of offering less energy dense swaps.

The impact on foods purchased will be measured using energy density. In experimental studies, higher energy density is linked to excess consumption and weight gain [5] and in observational studies shows clear links to weight gain and the risk of obesity [6], making reductions in energy density a clinically valuable outcome. Energy density also facilitates comparison across products irrespective of unit of purchase, unlike total energy purchased, and in pilot data obtained using the current platform, it showed relatively low variability for foods purchased.

The current study also explored the differential effectiveness of where the swaps were offered, and of asking consent to offer swaps. Firstly, swaps offered at the point of selection as opposed to at checkout were hypothesized to be more accepted and therefore more effective (Hypothesis 1) since at point of choice the participant is less committed to the original choice and the swap may represent less of a loss [7]. Secondly, swaps offered with the participant’s consent were hypothesized to be more accepted than imposed swaps (Hypothesis 2) since the imposition of a swap may be construed as reducing personal autonomy thus reducing the acceptability of the intervention [8]. Moreover, asking participants to consent to swaps may bolster a personal commitment to a healthy eating goal [9] and evoke higher acceptance of the swaps. Lastly since swaps offered must be accepted by the participant to impact on their purchase, accepting more swaps is anticipated to reduce the energy density of the final basket of purchases (Hypothesis 3).

Finally the current study aimed to measure whether the intervention leads to unintended consequences through self-licensing effects previously shown in other food choice studies [9–12] whereby individuals choose less healthy and more hedonic foods and justify (license) these actions on the basis of their compliance with a health-promoting intervention [13].

The current study used an experimental online supermarket platform. The paper first describes development of the platform, where several testing methods and outcome variables were explored. It then describes how these data informed the experimental design for the main study in which the effectiveness of swaps within the experimental online supermarket was assessed.

## Method

### Platform development

Before testing the effect of an intervention, the testing platform and methods were piloted. A website was built to emulate an online supermarket website for assessing the effectiveness of food purchase interventions (www.woodssupermarket.co.uk developed by Cauldron, UK http://cauldron.sc/clients#woods). The participant-facing website was built to give the appearance and functionality of an online supermarket website (browsing, search, unique product pages, trolley and checkout) but did not take payment or arrange delivery of any foods. The food database was a copy of the full range of foods and drinks from a real UK grocery retailer (Tesco.com API, February 2012, circa 11,000 products), supplemented with nutrient composition per 100 g (from the food labels collected by Kantar WorldPanel, or from databases for common foods supplied by MRC Human Nutrition Research [14]). The platform automatically collects all activity during the shopping visit as well as details of the foods bought such as their size, quantity, price and nutrient data. The researcher-facing website permits the appearance and functionality of the participant-facing website to be altered to implement a test intervention.

A pilot study within the default version of the website was conducted to inform study design questions (shopping task, outcome measure, sample size) for the current study. Of the 144 participants from a local volunteer panel who registered to take part, 85 completed two shopping sessions a week apart as instructed. Test-retest reliability and between-participant variability were assessed for four shopping tasks. Since over two thirds of people use a shopping list when shopping [15, 16], three lists were used in the pilot testing differing in the magnitude of choice available to the participant eg. “a loaf of bread” or “something to eat now”. To tie in with previous research using experimental shopping platforms that used broader shopping tasks [17–19], the fourth task asked participants to buy food for a family meal (see Additional file 1: Table S1 for details).

Participant’s choices differed from one week to the next and all four tasks showed low levels of test-retest reliability, both in terms of products bought and in terms of nutrient outcomes (intra-class correlation coefficients (ICC) < 0.5) (see Additional file 2: Table S2 for full data). Reliability was higher in response to two of the list tasks when foods were quantified in terms of proportion of healthier or less-healthy foods (ICC from 0.51 to 0.72). All four tasks also showed high levels of between-participant variability, both in terms of products bought and in terms of nutrient outcomes (coefficients of variance (CV) > 0.2) (see Additional file 2: Table S2 for full data). The lowest variability occurred when foods were quantified in terms of energy density (CV = 0.16).

Since none of the tasks was consistently better than the others in terms of test-retest reliability or between-subject variability, the shopping task used in the current study incorporated a mix of both targeted and open-ended list items. In order to maximise power to detect an effect of the intervention in the main study, variability of the outcome measure was minimised by extending the task to include more food items and by using energy density as the primary outcome - the nutrient outcome showing the lowest coefficient of variance.

### Subjects

A nationally representative sample of adult participants was recruited from a national research agency panel (www.ResearchNow.com, for demographics see Additional file 3: Table S3). This study received approval from the institution research ethics committee (University of Cambridge PRE.2012.069).

Participants took part in the study over the internet during February 2014, and both participant and experimenter were blind to group allocation. Invited participants were directed to a screening website where they gave informed consent to take part in the study, answered screening questions, provided demographic measures and reported prior online shopping experience.

Participants were screened to ensure they were responsible for at least half of their household grocery shopping using the shopping responsibility measure from the Food and You Survey [20], and that they purchased a least 75 % of the items on the shopping list at least once a year. Participants were screened to ensure they were attending to the questions as is typical in online testing protocols [21] (see Additional file 4: Additional methods for measures and Additional file 5: Table S4 for latent demand data).

Participants were asked to give their age, gender, height and weight in units of their choice for BMI calculation and two measures of SES: their highest educational qualification (UK census levels), and residential postcode to determine the quintile of the Index of Multiple Deprivation (area-level UK government index).

### Procedure

Following screening, eligible participants were randomly allocated by the study website (www.qualtrics.com) to one of five conditions: No intervention; Consent swap at point of product selection; Consent swap at point of checkout; Imposed swap at point of product selection; and Imposed swap at point of checkout.

Participants were then directed to a website which introduced the online shopping task as follows: “We would like you to do a shopping task. To do this, we have an online supermarket website for you to use. This is not a real commercial site, and you will not be asked to spend your own money or enter any personal details. When doing the shopping task, please choose foods you have normally bought in the recent past, or would probably choose given the range available, and don’t take too much time over your choices. You will also be given a budget. The budget is a guideline amount to spend – you do not need to spend it all, and although the website will let you overspend, please try to choose carefully to stay within the budget.” Participants were then provided with the shopping task and a link to the supermarket platform.

The current study employed a list task to ensure that the number, weight and cost of the basket of purchases were kept similar across participants. Individual items on the list were chosen to match those used in the platform development testing, and to maximize the opportunity for the purchase of items for which SES variability has previously been observed (e.g. high versus low fiber bread, confectionery, cheese [22]). Since prior work has suggested it is inappropriate to combine food and drinks when calculating energy density, the list included only food items [23]. The budget was chosen based on the amount spent during platform development testing in response to a similar 11-item list (median £19.80, IQR £17.38 to £23.58), with all participants given the same budget. The wording, items and budget allocation were as follows:

Please can you buy the following items: Budget: £25

- A loaf of bread (approx. 800 g loaf)
- Soup for a light meal for one
- A packet of ready-to-eat meat or fish, or a packet of cheese
- 1 pack of sweet biscuits
- Crisps or savoury snacks for 6 people
- Chilled dairy dessert for 6 people
- Meat/fish/vegetarian alternative for a roast lunch (approx. 500 g)
- 500 g pack of pasta/rice/couscous/polenta or other starchy food
- A side dish to have with a main meal
- A snack to have between meals
- A sandwich filling
- A treat for you to eat now

### Intervention

Participants in the four intervention groups were offered the option to select lower energy density foods while they shopped. For each food item selected to add to the shopping basket (base food), the platform automatically identified all the possible alternative foods that met the following criteria: within the same product category as the base food (as defined by the ‘shelf’ location used by the retailer from which the food database originated, see www.woodssupermarket.co.uk), between 90 % and 110 % of the weight and price of the base food, and with an energy density less than the base food by at least 100 kJ/100 g. Where more than two foods met the criteria, two were randomly selected from those available; where only one or two foods met the criteria, all were presented; and where no foods met the criteria, no swap was offered.

Some representative examples of swaps generated in the current study are as follows: a loaf of Hovis Country Granary bread (800 g, £1.34, 998 kJ/100 g) in exchange for a loaf of Warburtons Seeded Batch bread (800 g, £1.40, 1262 kJ/100 g); Tesco Black Forest Trifles (3x150g, £2.00, 590 kJ/100 g) in exchange for a Tesco Family Tiramisu (500 g, £1.98, 1110 kJ/100 g); Wholewheat Fusilli Pasta (500 g, £0.89, 1360 kJ/100 g) in exchange for Tagliatelle (500 g, £0.98, 1505 kJ/100 g); Cadbury Crunchie bar (40 g, £0.57, 1950 kJ/100 g) in exchange for Maltesers Standard Bag (37 g, £0.54, 2112 kJ/100 g).

A pop-up window displayed when logging-in to the platform introduced the swaps to intervention group participants: “The website you are about to use is designed to help you buy food with fewer calories. It will suggest alternatives to your choices that: have fewer calories per gram, and are from the same food category.”

The four intervention groups differed in terms of how the swaps were implemented following a two-by-two factorial design. Swaps were either offered one at a time as soon as each base food item was added to the basket (selection), or were offered all at once at the end of the shopping task (checkout). The inclusion of swaps within the site was framed in the introductory message as either something participants had consented to (Consented: “Please shop using the website and give us feedback.”) or as something imposed on participants (Imposed: “Your task is to shop using the website and give us feedback.”).

### Measures

All food measures excluded foods classed as ‘off-list’ items due to their extreme energy densities or their use in food preparation in small quantities. ‘Off-list’ items were all drinks (soft drinks, water, fruit juices, smoothies, milk, alcohol, tea, coffee and hot chocolate drinks) and cooking ingredients (butter, oils, margarine and other fats, salt, stock cubes, sugar or sweeteners, vinegar, flour).

A prompt offering a swap could elicit one of three responses from the participant: the base food could be added to the basket, an alternative food could be added to the basket or the participant could click ‘back’, not add anything to the basket and return to the previous screen. Only swaps which resulted in a food being added to the basket were included in the analysis, with each swap scored as either accepted (the alternative food was added to the basket) or refused (the base food was added to the basket).

### Proportion of swaps accepted

The number of accepted swaps as a proportion of the total number of accepted and refused swaps.

### Energy density gain per swap

The energy density difference between the base food and the alternative food (kJ/100 g).

### Bought energy density

The total energy density (kJ/100 g) of the basket of foods purchased by the participant.

### Base energy density

The energy density (kJ/100 g) of the basket of original foods chosen, i.e. had the platform not offered the opportunity to swap to an alternative.

### Potential energy density

The energy density (kJ/100 g) of the basket of foods that could potentially be purchased, i.e. had the participant accepted all swap alternatives offered by the platform.

### Treat item FSA nutrient profile

The FSA Nutrient Profile (FSA NP) [24] of the last list item (“A treat for you to eat now”). Lower scores indicate healthier foods.

### Supermarket rating

After completing the shopping task, all participants rated their shopping experience. “Overall, how would you rate your shopping experience using Woods?” with seven response options (excellent, very good, good, neutral, poor, very poor, terrible).

### Intervention acceptability

After completing the shopping task, participants in the four intervention groups indicated the acceptability of the swap intervention experienced. “The online supermarket you have just used offered you healthier foods as alternatives to some of the foods you originally chose. Is this something you would like to have when you do your usual shopping?” with eight response options (very strongly like, strongly like, somewhat like, indifferent, somewhat dislike, strongly dislike, very strongly dislike, I didn’t notice any alternatives being offered to me).

### Online shopping experience

Within the screening questionnaire all participants reported their prior online shopping experience for groceries and non-food items: “How often, on average over the past year, have you shopped online for food or groceries to be delivered to you (e.g. Tesco.com, Ocado.com, myspermarket.co.uk)?” and “How often, on average over the past year, have you shopped online for any non-food items to be delivered to you (e.g. books, clothes, electronics)?”, both scales with five response options (never or not in the last year/1-3 times in the last year/4-11 times in the last year/1-3 per month/once per week or more often).

### Secondary hypotheses

Self-licensing effects were hypothesized to result from the presence of a swaps intervention as captured by one of two measures: what foods participants freely chose before swaps were prompted (Base energy density) and the healthiness of the food chosen when invited to indulge (Treat FSA Nutrient Profile). Swaps at the point of selection were hypothesized to result in more self-licensing than at the point of checkout because of the greater visibility of the swaps interrupting the shopping exercise (Secondary Hypotheses 2). Imposed swaps were hypothesized to result in more self-licensing than consented swaps because consent bolsters personal commitment to a goal of healthy eating [9] which would mitigate a desire to indulge.

Demographic variables were predicted not to modulate the main effect of the intervention, as measured by proportion of swaps accepted, given the effectiveness of the intervention was not contingent on nutritional knowledge, education or on economic factors.

Accepted and refused swaps were compared in terms of the base food energy density, the base food category, and the magnitude of the change in energy density that the swap offers to capture whether one of these attributes was associated with greater swap acceptance.

Acceptability of the intervention was hypothesized to correlate with proportion of swaps accepted across participants in the four swap intervention groups, given accepting the swaps is an indicator of positive interaction with the intervention.

### Analysis

The data were cleaned to ensure only one visit per participant was analyzed. Participants’ food purchases were screened to ensure compliance with the shopping list task (see Additional file 4: Additional methods for further details).

A regression analysis tested whether the number of swaps offered was the same by intervention type. A logistic regression analysis tested the proportion of swaps accepted by intervention type. Both models used planned contrasts to test for an effect of point of swap and framing.

A regression analysis of the energy density of the basket of foods purchased by intervention group with planned contrasts tested for the effect of intervention versus control, point of swap and framing. A second model included the number of swaps accepted as a covariate to test for the mediating effect of the number of swaps accepted.

Self-licensing behavior in response to being presented with the intervention was assessed by analyzing the energy density of the base items selected prior to being offered swaps, and the FSA nutrient profile [24] of the last item on the shopping list “a treat for you to each now”. Both were analyzed using regression models of outcome measure by intervention type using planned contrasts (control vs intervention groups, point of swap, framing).

The acceptability of the intervention was assessed by looking at the rated shopping experience by intervention type using planned contrasts (control vs intervention groups, point of swap, framing), using a correlation analysis to see if acceptability of the intervention varied as a function of the proportion of swaps accepted.

Logistic regression was used to determine whether swap acceptances varied as a function of the ED gain offered by the swap or by food category using Chi Squared test.

The required sample size was calculated with a power of 0.8 and α of 0.05 (G*Power, version 3.1.5) to detect a small effect size (Cohen’s d = 0.2) or a 3 % difference in energy density assuming a mean energy density of 937 (139) kJ/100 g (based on the pilot data above). This gives a total sample size of 954 participants to detect a difference between the control group and the four intervention groups.

## Results

### Response screening

**Fig. 1:**
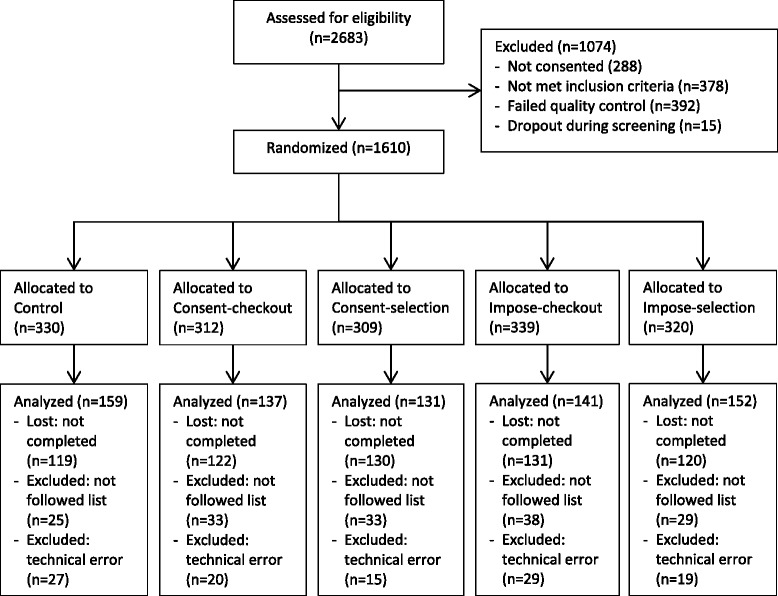
Consort diagram of participation through the study

Of the 2683 unique participants who were assessed for eligibility, 1610 were randomized to one of the five study groups (See Fig. 1). Overall, 622 (39 %) did not complete the testing procedure for unknown reasons, and 988 completed testing. A further 110 had to be excluded due to a technical error, and 158 completed testing but did not fully comply with the task (e.g. bought fewer than 10 items, bought 3 or more off-list items, both indicative of not following the list task) and their data were not included in the final analysis, leaving 720 participants. These exclusion and loss rates were not significantly different across the five randomized groups (*Χ*^2^(12) = 10.92, p = 0.464).

### Primary outcome

Participants purchased a mean (SD) of 12.36 (1.26) foods, totaling 49.96 MJ (12.45) and weighing 4.99 kg (1.42), with an energy density of 1019 kJ per 100 g (160).

Participants in the intervention groups responded to a mean of 4.08 (SD = 1.68) prompts to swap a food they had selected. The potential energy density of the basket of food purchased by the intervention groups had all swaps been accepted was significantly lower than the food purchased by the control group (effect size (95 % CI): −83 kJ/100 g (−110 – -56), p = <0.0001). There were no differences between intervention groups in the number of swaps offered or the potential energy density of the basket if all swaps were accepted (Table 1).

**Table 1:** Number of swaps offered, potential basket energy density, number of swaps accepted and bought basket energy density by participant group

	Group					Planned contrast effect sizes (95 % CI)		
	Control (n = 159)	Consent-checkout (n = 137)	Consent-selection (n = 131)	Imposed-checkout (n = 141)	Imposed-selection (n = 152)	Intervention vs Control	Consent vs Imposed	Selection vs Checkout
N Swaps offered	-	4.17	3.96	4.01	4.17	-	−0.003 (−0.04 – 0.03)	−0.003 (−0.04 – 0.03)
Potential basket ED (kJ/100 g)	1037.6	969.6	962.9	939.1	947.1	−82.9*** (−110.1 – -55.8)	23.1 (−2.4 – 48.7)	0.6 (−24.9 – 26.2)
N swaps accepted (median, OR)	-	0 (0–1)	1 (0–2)	0 (0–1)	1 (0–2)	-	0.977 (0.88 – 1.08)	1.224 (1.11 – 1.35)
Bought basket ED (kJ/100 g)	1037.6	1026.5	1023.3	997.0	1007.2	−24.1 (4.04 – -52.23)	22.8 (−3.69 – 49.26)	3.53 (−22.94 – 20.01)

***: *p* < 0.001

A median of one swap (inter-quartile range 0 to 2) was accepted and resulted in the alternative item being added to the basket. Four (0.7 %) of the intervention participants were not offered any swaps and 264 (47.1 %) of the intervention participants did not accept any swaps they were offered. Swaps offered at selection were significantly more likely to be accepted than swaps offered at checkout (OR (95 % CI): 1.224 (1.11 – 1.35), p = <0.0001) (Table 1). Consented swaps were no more likely to be accepted than imposed swaps (OR (95 % CI): 0.977 (0.88 – 1.08), p = 0.649) (Table 1).

**Fig. 2:**
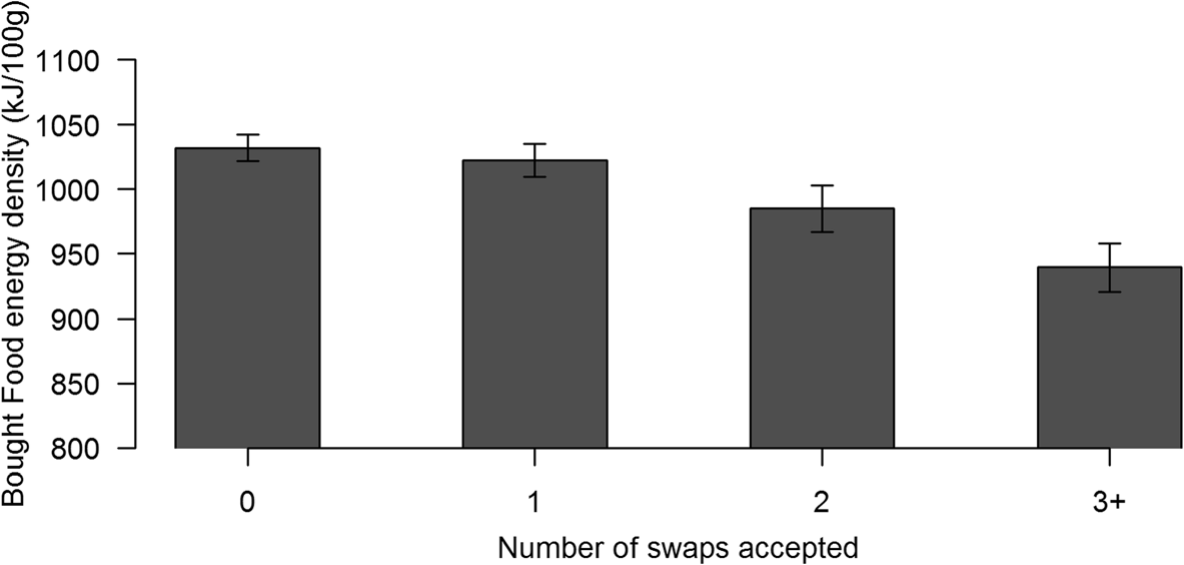
Purchased food energy density as a function of number of swaps accepted for participants offered swaps (all intervention groups combined). (Error bars indicate SEM)

The energy density of the food purchased by the intervention groups was not significantly different to the food purchased by the control group, and did not differ by how the intervention was implemented (Table 1). Although a larger proportion of participants did not accept any swaps offered to them, participants that did accept the swaps did benefit as indicated by an association between the number of swaps accepted and a reduction in the energy density of food purchased (effect size per swap accepted (95 % CI): −24 kJ/100 g (−34.6 – -13.8), *p* < 0.0001) (Fig. 2).

### Secondary outcomes

#### Self-licensing behavior

Neither measure testing for possible self-licensing behavior in response to the intervention showed any significant difference between groups. Regression analysis of the energy density of the base food (the initial selections made prior to swaps being offered) revealed no significant differences for the intervention groups compared with the control group (effect size (95 % CI): 14 kJ/100 g (−32 – 60), p = 0.551), for swaps framed as consented compared with swaps framed as imposed (effect size (95 % CI): 38 kJ/100 g (−15 – 90), p = 0.159) or swaps offered at checkout compared with swaps offered at selection (effect size (95 % CI): −14 kJ/100 g (−67 – 38), p = 0.588).

Analysis of the FSA nutrient profile score for the last item purchased (“A treat for you to eat now” analyzed for 663 out of the 684 participants) revealed no significant differences for the intervention groups compared with the control group (effect size (95 % CI): 0.62 (−1.87-3.11), p = 0.626), for swaps framed as consented compared with swaps framed as imposed (effect size (95 % CI): 0.155 (−2.67-2.98), p = 0.915) or swaps offered at checkout compared with swaps offered at selection (effect size (95 % CI): −1.55 (−4.38-0.13), p = 0.282).

### Demographics

Collapsing by intervention group, female participants and less deprived participants (as indicated by higher IMD quintiles) were more likely to accept swaps. There was no effect of education on the acceptance of swaps (Table 2).

**Table 2:** Logistic regression of swap acceptance rate by demographic variables

	OR (95 % CI)	*p*
(Intercept)	0.12 (0.06-0.23)	<0.0001
Age (standardized)	1.06 (0.95-1.17)	0.280
BMI (ref lean)		
Overweight	0.95 (0.63-1.47)	0.817
Obese	1.13 (0.75-1.74)	0.584
Gender (ref male)	1.31 (1.06-1.61)	0.012
Education (per Education level)	1.03 (0.96-1.10)	0.384
IMD quintile (per quintile)	1.11 (1.05-1.18)	0.0004

### Swaps description

The mean energy density of the base product for the accepted swaps was 1400 kJ/100 g (SD 625), and not significantly different from that for refused swaps (effect size (95 % CI): 1.9 kJ/100 g (−67.4 – 71.3), p = 0.953). However swaps offering larger reductions in energy density resulted in lower levels of acceptance (OR per standard deviation of swap ED gain (95 % CI): 0.81 (0.71 – 0.93), p = 0.003). The current task was not able to separate foods purchased by shopping list item since participant purchased the list in one visit to the supermarket, though an analysis based on supermarket food category was conducted to explore whether swap acceptance differed by food type. Different levels of swap acceptance were seen within different food categories (*Χ*^2^(11) = 66.63, *p* < 0.0001) (Table 3) with the greatest acceptance of swaps within the pasta, rice and noodles category, and lowest for fresh poultry. Different mean reductions in energy density for accepted swaps were seen for different food categories, with the largest reduction for Cooked and Continental Meats (325 kJ/100 g).

**Table 3:** Swap acceptance rate and accepted swap mean energy density reduction (difference between base product and swap product) by food category

Category (N swaps)	% Accepted	Mean ED reduction kJ/100 g (s.d.)
Crisps Snacks & Nuts (478)	19 % *	240 (96)
Pasta Rice & Noodles (269)	36 % ***	195 (214)
Cooked & Continental Meats (226)	31 % **	325 (183)
Biscuits (191)	18 %	263 (160)
Tins Cans & Packets (187)	19 %	148 (78)
Dairy Eggs & Cheese (140)	27 %	291 (176)
Chocolate & Sweets (114)	25 %	207 (173)
Fresh Poultry (114)	6 % ***	249 (59)
Fresh Meat (91)	14 % *	292 (77)
Chilled Desserts (79)	19 %	190 (132)
Bread (58)	31 %	154 (48)
Other (339)	25 %	291 (236)

Significance calculated from standardized Pearson Residuals. *: *p* < 0.05, **: *p* < 0.01, ***: *p* < 0.001

### Acceptability

The rating given to the overall shopping experience was significantly better for participants in the intervention groups than in the control group (effect size (95 % CI): 0.356 (0.083-0.629), p = 0.011), and significantly better for consented swaps than imposed swaps (effect size (95 % CI): 0.326 (0.017-0.634), p = 0.039), with no difference in point of swap (effect size (95 % CI): −0.010 (−0.318-0.298), p = 0.712). The acceptability rating for the intervention was significantly correlated with the number of swaps accepted (effect size per swap accepted (95 % CI): 0.562 (0.469-0.658), *p* < 0001).

## Discussion

The current study showed that offering lower energy density swaps within specific product categories (i.e. suggesting lower ED biscuits instead of higher ED biscuits) did not significantly lower the energy density of the basket of foods purchased. While the automated algorithm identified swaps for a third of the foods purchased (4.1 swaps out of 12.4 foods purchased), participants accepted a median of only one swap. Had all the swaps been accepted, there was the potential to improve the basket energy density by up to 82.9 kJ/100 g, equivalent to a net reduction of 2811 kJ over the whole shopping basket or 5.9 % less energy purchased. Likelihood of acceptance was increased when swaps were offered at the point of selection compared to checkout (OR (95 % CI) = 1.224 (1.11 – 1.35)) (Hypothesis 1), but was unaffected by explicit consent (Hypothesis 2). Purchased food ED was unaffected by point of swap or explicit consent. Many (47 %) participants refused all the swaps offered to them, and as a function did not benefit from the intervention. The participants that did accept the swaps did benefit: each swap accepted was associated with a reduction in purchased food ED of 24 kJ/100 g, equivalent to 1173 kJ less or a reduction of 2.3 % in energy purchased per swap accepted (Hypothesis 3).

Analysis of the rate of swap acceptance showed differences between individuals and food categories. Female participants and less deprived participants (higher IMD quintile) were more likely to accept the swaps offered to them, suggesting that these subgroups of the population may be more likely to benefit from such an intervention. Pasta and rice, and sandwich fillings were associated with higher rates of swap acceptance, whereas raw meats and savory snacks were associated with lower rates of swaps acceptance. Why so many participants did not accept the swaps is a question for future research. It seems likely that individual differences in how foods are perceived or chosen would play a role; for instance the strength of preference for one food over alternatives from the same category, the extent to which food choices are habitual, or the extent to which the swap is viewed as functionally equivalent with the original choice, an issue perhaps more likely to arise in heterogeneous food categories like raw meat. It may also be that framing the swaps as offering lower calorie alternatives was off-putting, and that framing the swap as offering a more popular or cheaper option might be more appealing.

We also explored whether offering swaps led to self-licensing of other food purchases. There was no significant difference between groups in the ED of the initial foods selected or the nutrient profile of the final item on the list (“a treat to eat now”). This suggests that the swap interventions did not elicit self-licensing effects and alter the baseline food choices made by participants.

In terms of acceptability, the current study gives no indication that the presence of swaps was detrimental to the shopping experience. In fact the reverse was found - participants who engaged more with the swaps by accepting more of them reported a more positive shopping experience.

It is notable that the current study finds effects that are much smaller than the 10 % reduction in saturated fat purchased reported in a previous study [4]. They did not report on the uptake of swaps which makes it hard to determine what accounts for the difference in outcome. However, there are a number of key differences in design between the studies. Huang et al. targeted a single nutrient (saturated fat), and pre-specified swaps were selected to offer the largest possible reduction in saturated fat content from anywhere within the store (e.g. yogurt instead of cream). By contrast, the current study targeted energy density, a composite of multiple nutrients, and swaps were limited to within a category. While necessary to increase the likelihood that swaps were functionally appropriate, this process meant swaps offered in the current study almost certainly represented smaller gains than those which can be achieved with cross-category swaps.

The current study was conducted within an experimental online testing platform that emulates an online grocery store. Translational research platforms such as this [17–19, 25, 26] have advantages over controlled laboratory studies and real world shopping research for exploring food purchasing. The use of an online platform rather than a laboratory setting makes it feasible to include and manipulate some of the defining aspects of the grocery shopping environment; such as the large range of foods and brands, the location of foods within the store, and the inclusion of price promotions and marketing images. The use of an experimental platform rather than a grocery store means greater control of the shopping task, and hence lower between-subject variability and smaller sample sizes to find effects. These translational platforms can be used to generate, at relatively low cost, preliminary data on the effectiveness of an intervention that can then be used to engage retailers in further testing in a real-world setting. While the use of a translational platform has a number of limitations (see below), it generates data that can inform future testing and implementation of an intervention design to change health across a population.

Pilot testing during the development of the platform showed low test-retest reliability and high between-subject variability, despite efforts to minimize both with the use of relatively tightly defined shopping tasks. The reason for this is unclear. Real world shopping behavior would be expected to vary with household size, budget and shopping lists, but these were controlled for in the current study. Other possible sources of variability are individual trait differences in terms of food preferences, liking, familiarity, and food goals (e.g. taste, health) [27], as well as individual state differences, such as hunger, tiredness, or the distractors while completing the shopping exercise; none of which were controlled in the study. In spite of this, the pilot testing allowed us to refine the protocol for the study in relation to both the task and the outcome measure.

This study had a number of strengths. Giving all participants the same shopping task ensured participants purchased the same number of items and reduced the variability of the outcome measure, resulting in greater power to find effects than had each participant purchased to their own list. Using a bespoke online shopping platform rather than collaborating with a real retailer also permitted trialing of a number of different implementations of a swaps intervention. Using a grocery listing supplied by a real UK grocery retailer maximized the opportunity for participants choose foods that were liked and familiar, and minimized the possibility that participants were forced into satisfying the task demands with the options available. The study was also able to test a swaps intervention when automated across several thousand foods – a challenge for scaling laboratory interventions to a supermarket environment. Finally, conducting the study online enabled a broader range of socio-economic groups to be recruited without interacting with the researchers, something that is not feasible with laboratory testing.

However, this study design brings a number of limitations. This study, as with other online studies [28], has low completion rates perhaps reflecting interruptions or distractions as participants took part in their own home. The shopping task used was specified by the experimenter, although possible demand effects were mitigated by recruiting participants with latent demand for the list items. Given the swap identification was automated, some suggestions may have been functionally inappropriate. While undesirable, the criteria adopted in the current study ensured this happened infrequently, and the automation ensured a pragmatic solution to the issue of identifying appropriate swaps for all 11,000 products for over 1000 participant shopping visits. Finally, the behavior captured here provides a measure of expressed preference within a real-world-like environment, but does not represent real food purchasing. While this means the study has less external validity than a study conducted within a real store, its design brings strengths over studies in real stores and is intended to inform future studies that have greater external validity.

Interventions to improve the healthiness of food purchases that are solely implemented within online supermarkets are unlikely to be effective population-wide interventions. A relatively narrow and unrepresentative range of individuals from the UK population shop for food online (9 % of the population overall, rising to over 20 % for households with children or a high income [20]). In addition there are known differences between the healthiness of foods purchased for home delivery when compared with in-store purchases [29]. A swap intervention within an online grocery shopping website therefore represents only one of a range of potential public health tools to decrease the energy density of food purchases and later consumption, and may be more effective when used in concert with other interventions such as pricing manipulations, labelling and public education campaigns.

The real value the current research is the use of an experimental online testing platform to generate useful data to inform public health intervention decisions at relatively low cost and without retailer collaboration. These data demonstrate that offering swaps within-category has a limited impact on the overall ED of purchased food; firstly because the effect was limited by a modest possible gain had all the swaps been accepted, and secondly because none or only a few swaps were accepted. Individuals who accepted the swaps did reduce the energy density of the food purchased suggesting increasing acceptance rates could improve the impact of swaps. Think-aloud studies [30] could provide an indication of whether and how this might be done, and experimental studies could show whether swap acceptance rates can be improved by framing the swap as more popular or cheaper rather than healthier. Implementing swaps to offer alternatives across category, e.g. fruit instead of biscuits, may promise larger ED gains but would be less amenable to automation and probably have lower acceptance rates since swaps offering larger reductions in energy density we associated with lower acceptance rates. In short, swaps may be beneficial for some subgroups that accept them and no unintended consequences were observed, but offer limited population-wide benefits as a sole intervention.

## Additional files

Additional file 1: Table S1. Platform development method. Additional file 2: Table S2. Platform development results. Additional file 3: Table S3. Participant Demographics. Additional file 4:
**Additional methods (quality control items and screening for compliance).**
Additional file 5: Table S4. Participant Latent demand and online shopping experience).

## Abbreviations

ED: Energy density
