# Supplementary material for: Offering within-category food swaps to reduce energy density of food purchases: a study using an experimental online supermarket
Source: Int J Behav Nutr Phys Act. 2015 Jun 25;12:85. doi: 10.1186/s12966-015-0241-1 (PMC4488046; doi:10.1186/s12966-015-0241-1)
Supplement: Additional file 1: Table S1. — Platform development method. [file 12966_2015_241_MOESM1_ESM.pdf]

Supplementary Table 1: Platform development method

	List-Repeating	List-Matched	List-Category	Meal
**Please can you buy the following items:**	• A loaf of bread (approx. 800g loaf)<br>• Soup for a light evening meal<br>• A packet of ready-to-eat meat or fish, or a packet of cheese<br>• 1 pack of sweet biscuits	• *A large bag*/ **A multipack of 6 small bags** of crisps for sharing.<br>• *Frozen*/**Chilled** dairy dessert for 6 people<br>• Meat or fish *for cooking on a barbecue*/ **a roast lunch** (approx. 500g)<br>• Non-alcoholic drinks for *on-the-go*/ **a meal**	• A side dish to have with a main meal<br>• A snack to have between meals<br>• A sandwich filling	Using the website, please choose all the food and drink you would need for a regular weekday main meal for a family of 4 (2 adults and 2 older teenagers). You don't need to buy any items that you might need for preparing the food like oil, salt or pepper.
**Budget:**	£7	£8	£6	£10
