# Supplementary material for: Offering within-category food swaps to reduce energy density of food purchases: a study using an experimental online supermarket
Source: Int J Behav Nutr Phys Act. 2015 Jun 25;12:85. doi: 10.1186/s12966-015-0241-1 (PMC4488046; doi:10.1186/s12966-015-0241-1)
Supplement: Additional file 2: Table S2. — Platform development results. [file 12966_2015_241_MOESM2_ESM.pdf]

Supplementary Table 2: Platform development results

| | List-Repeating | | | List-Matched | | | List-Category | | | Meal | | |
|---|---|---|---|---|---|---|---|---|---|---|---|---|
| **Minutes taken/ median (IQR)** | 6.0 (4.1 – 9.3) | | | 8.2 (5.7 – 11.2) | | | 4.2 (2.6 – 6.2) | | | 7.7 (4.9 – 11.1) | | |
| **Product outcomes** | | | | | | | | | | | | |
| **N participants buying 2+ foods bought by other participants (out of 85)** | 39 | | | 45 | | | 2 | | | 52 | | |
| **N participants buying 2+ foods (2+ shelves) in both sessions out of 85** | 13 (78) | | | 2 (39) | | | 7 (27) | | | 18 (59) | | |
| | Mean | CV | ICC | Mean | CV | ICC | Mean | CV | ICC | Mean | CV | ICC |
| **Nutrient outcomes** | | | | | | | | | | | | |
| **Total Energy (MJ)** | 18.19 | 0.21 | 0.39 | 11.16 | 0.39 | 0.21 | 6.31 | 0.57 | 0.44 | 19.48 | 0.52 | 0.23 |
| **Energy Density* (kJ/100g)** | 993.0 | 0.16 | 0.27 | 849.3 | 0.29 | 0.08 | 709.4 | 0.53 | 0.30 | 523.5 | 0.45 | 0.27 |
| **SFA (% energy)** | 12.60 | 0.44 | 0.21 | 12.04 | 0.54 | 0.00 | 13.40 | 0.70 | 0.31 | 12.67 | 0.57 | 0.10 |
| **Sugar (% energy)** | 10.86 | 0.35 | 0.36 | 19.78 | 0.43 | 0.00 | 23.33 | 0.68 | 0.18 | 13.69 | 0.72 | 0.40 |
| **Fibre (g per MJ)** | 3.40 | 0.33 | 0.34 | 0.94 | 0.65 | 0.06 | 4.24 | 0.57 | 0.43 | 3.58 | 0.57 | 0.35 |
| **Sodium (mg per MJ)** | 360.6 | 0.29 | 0.23 | 281.5 | 0.43 | 0.00 | 399.7 | 0.68 | 0.23 | 285.3 | 0.66 | 0.08 |
| **Nutrient score (35) (weighted by energy)** | 4.90 | n/a | 0.27 | 5.46 | n/a | 0.00 | 2.73 | n/a | 0.38 | 1.07 | n/a | 0.02 |
| **Food Group outcomes** | | | | | | | | | | | | |
| **Fruit & Vegetables (% weight)** | 0.48† | 0.53 | 0.36 | 2.38† | 0.32 | 0.00 | 52.38 | 0.45 | 0.52 | 41.78 | 0.39 | 0.52 |
| **Healthier (% products)** | 21.45 | 0.34 | 0.70 | 14.73 | 0.49 | 0.03 | 40.98 | 0.48 | 0.66 | 62.61 | 0.25 | 0.38 |
| **Less healthy (% products)** | 46.16 | 0.27 | 0.52 | 44.77 | 0.28 | 0.00 | 27.59 | 0.53 | 0.51 | 17.92 | 0.44 | 0.04 |

***: Calculated for food only, excluding drinks.(25)**
**†: No fruit or vegetable items were included in these task**
