# Supplementary material for: Offering within-category food swaps to reduce energy density of food purchases: a study using an experimental online supermarket
Source: Int J Behav Nutr Phys Act. 2015 Jun 25;12:85. doi: 10.1186/s12966-015-0241-1 (PMC4488046; doi:10.1186/s12966-015-0241-1)
Supplement: Additional file 3: Table S3. — Participant Demographics. [file 12966_2015_241_MOESM3_ESM.pdf]

Supplementary Table 3: Participant Demographics

		**All (n=720)**	**Control (n=159)**	**Consent-checkout (n=137)**	**Consent-selection (n=131)**	**Imposed-checkout (n=141)**	**Imposed-selection (n=152)**
BMI status	Lean (18 to <25)	42.2%	44.7%	36.5%	42.0%	50.4%	37.5%
	O'weight (25 to <30)	30.8%	31.4%	30.7%	32.1%	27.7%	32.2%
	Obese (30+)	26.8%	23.9%	32.8%	26.0%	22.0%	29.6%
Age	Mean (s.d.)	44.4 (14.13)	43.4 (14.38)	45.6 (14.49)	45.4 (14.43)	43.2 (13.37)	44.8 (13.98)
Gender	% female	62.2%	62.89%	65.69%	61.07%	65.25%	56.58%
Diet for weight loss/ maintenance	% dieting	34.7%	39.0%	35.8%	34.4%	31.9%	32.2%
Education	No quals.	4.7%	4.4%	5.1%	2.3%	3.5%	7.9%
	Up to 4 GCSE's	17.2%	19.5%	12.4%	20.6%	16.3%	17.1%
	Up to 1 A-level	20.4%	20.8%	22.6%	21.4%	16.3%	21.1%
	2+ A-levels	22.5%	26.4%	18.2%	24.4%	25.5%	17.8%
	First Degree	23.8%	20.1%	27.0%	19.8%	28.4%	23.7%
	Further Degree	11.4%	8.8%	14.6%	11.5%	9.9%	12.5%
IMD Quintiles	Not available*	16.8%	13.2%	22.6%	16.8%	15.6%	16.4%
	1	15.4%	13.8%	13.1%	18.3%	12.8%	19.1%
	2	18.3%	20.8%	16.1%	22.1%	15.6%	17.1%
	3	17.8%	22.6%	20.4%	11.5%	17.7%	15.8%
	4	16.3%	13.8%	14.6%	12.2%	24.8%	15.8%
	5	15.4%	15.7%	13.1%	19.1%	13.5%	15.8%

*Participants from Scotland, Wales or Northern Ireland
