# Supplementary material for: Offering within-category food swaps to reduce energy density of food purchases: a study using an experimental online supermarket
Source: Int J Behav Nutr Phys Act. 2015 Jun 25;12:85. doi: 10.1186/s12966-015-0241-1 (PMC4488046; doi:10.1186/s12966-015-0241-1)
Supplement: Additional file 4: — Additional methods (quality control items and screening for compliance). [file 12966_2015_241_MOESM4_ESM.pdf]

Additional methods

Quality Control items:

Two measures were used: “Do you sleep more than an hour (60 minutes) in total per night?” with responses on a 7-point scale from “1 (never)” to “7 (always)”; and “How often have you been to every country in the world (all 196 of them)” with seven response items - Never/ Not in the past year/ 1-3 times a year/ 4-11 times a year/ 1-3 times a month/ Once a week/ More than once a week. Participants were judged to be giving careless responses and were excluded if they gave any responses other than always to the sleep question (6 or 7) and never or not in the past year to the travel question (1 or 2).

Screening for compliance with the shopping task:

All purchases made by each participant were labelled as fulfilling one of the twelve shopping list items, or as being items unsolicited by the list (see below and measures). Participants who purchased foods that covered fewer than 10 of the list items or that included more than two ‘off-list’ items were coded as not complying with the shopping list task. These criteria were chosen on the understanding that a participant meeting ten or more of the shopping list items and only one or two off-list items showed intent to complete the task as requested. All outcome measures were based on the foods in the shopping basket when the participants ‘checked-out’ of the shopping website, after excluding ‘off-list’ foods (see measures), including foods that were not assigned as fulfilling a list item.

The process of assigning foods to a list item or as being ‘off-list’ was initially automated: foods from categories that clearly fulfilled one list item were assigned to that list item (e.g. bread, tinned or chilled soup, biscuits, crisps, dairy items, rice or pasta), while foods from categories that the shopping list did not require the purchase of were labelled ‘off-list’ (see measures for definition).

Following this, each individual’s food purchases, listed in the order in which they were added to the basket, were manually checked for the list-assignment and to code any remaining foods. For unassigned items, the product description and the order in which items were added to the basket were used to assign foods to list items. Where neither gave an indication of list membership, foods were left unassigned. The ‘treat’ item was assumed to be the last item added to the basket that was ready-to-eat.
