# Supplementary material for: Offering within-category food swaps to reduce energy density of food purchases: a study using an experimental online supermarket
Source: Int J Behav Nutr Phys Act. 2015 Jun 25;12:85. doi: 10.1186/s12966-015-0241-1 (PMC4488046; doi:10.1186/s12966-015-0241-1)
Supplement: Additional file 5: Table S4. — Participant Latent demand and online shopping experience). [file 12966_2015_241_MOESM5_ESM.pdf]

Supplementary Table 4: Participant Latent demand and online shopping experience

Study Participants (n=720)	Never or not in the last year	1-3 times in the last year	4-11 times in the last year	1-3 times a month	At least once a week
Online Shopping – food	47.5%	17.8%	16.8%	11.7%	6.3%
Online shopping – non food	3.5%	9.6%	42.2%	38.3%	6.4%
Latent purchasing demand					
Bread	0.3%	1.4%	3.3%	12.1%	82.9%
Soup	5.4%	10.4%	17.8%	34.2%	32.2%
Ready-to-eat meat or fish or cheese	1.8%	2.5%	6.9%	31.5%	57.2%
Sweet biscuits	1.3%	7.4%	14.9%	33.9%	42.6%
Crisps or savory snack	1.0%	5.6%	13.5%	31.8%	48.2%
Dairy dessert	3.2%	9.4%	14.6%	26.5%	46.3%
Meat or fish for roast lunch	1.1%	6.0%	10.6%	21.9%	60.4%
Pasta/Rice/couscous or other starch	3.2%	9.4%	14.6%	26.5%	46.3%
A side dish	1.1%	6.0%	10.6%	21.9%	60.4%
A snack for between meals	1.1%	4.2%	10.6%	19.2%	65.0%
A sandwich filling	7.4%	8.8%	13.5%	19.9%	50.6%
A treat for you to eat straight away	1.1%	8.5%	13.2%	22.9%	54.3%
